# Reduced quality of life in myasthenia gravis patients: A study on 185 patients from China

**DOI:** 10.3389/fneur.2022.1072861

**Published:** 2023-01-12

**Authors:** Xuan Wu, Run Yun Li, Xiao Bin Ye, Ning Wang

**Affiliations:** ^1^Department of Neurology, The First Affiliated Hospital of Fujian Medical University, Fuzhou, China; ^2^Department of Neurology and Institute of Neurology, The First Affiliated Hospital of Fujian Medical University, Fuzhou, China

**Keywords:** myasthenia gravis, quality of life, activities of daily living, Myasthenia Gravis Foundation of America, MG-QOL15

## Abstract

**Aims:**

To explore the quality of life (QOL) in patients with myasthenia gravis (MG) and factors associated with QOL.

**Methods:**

This observational study included patients with MG diagnosed at the First Affiliated Hospital of Fujian Medical University between January 2020 and March 2022. The QOL of patients was evaluated with the 15-item Myasthenia Gravis Quality of Life (MG-QOL15). Current MG severity was evaluated with MGFA grade, MG-ADL score, MGC score, and MGFA Postintervention Status. The data about gender, age of onset, subgroup, antibodies, age, duration, education, employment state, marital status, skeletal muscle affected, thymic histology, and current treatment methods of the patient were collected.

**Results:**

A total of 185 patients [72 males (38.9%), aged 45.2 years (14–77)] with MG were enrolled. Age at onset was 38.3 ± 17.9 years, and disease duration was 87.9 months (0–672). The median MG-QOL15 score was 12.5 (0–58). The item “have trouble using my eyes” was the highest scoring item in both ocular and generalized patients with MG. The MG-QOL15 score was significantly different among patients with OMG (9.2 ± 9.4, *n* = 63), GMG (9.0 ± 8.8, *n* = 22), and BMG (15.4 ± 14.2, *n* = 100) (*P* = 0.018). Patients with BMG had higher MG-QOL15 scores than OMG (*P* = 0.001) and GMG (*P* = 0.009), but there was no significant difference between OMG and GMG (*P* = 0.467). The MG-QOL15 score was significantly lower in patients who had undergone thymectomy (9.7 ± 9.8, *n* = 58) compared to those who had not (13.8 ± 13.4, *n* = 127, *P* = 0.022). MG-QOL15 score was significantly lower in patients who underwent thymectomy compared to those who did not (9.7 ± 9.8, *n* = 58 vs. 13.8 ± 13.4, *n* = 127, *P* = 0.022). MG-QOL15 score was different among MGFA grades (Remission: 5.2 ± 5.4, *n* = 41; I: 11.3 ± 10, *n* = 61; II: 11.6 ± 11.1, *n* = 40; III: 18.1 ± 12.1, *n* = 29; and IVa: 30.1 ± 20, *n* = 14, *P* < 0.001). There was no significant difference between patients in MGFA grade I and II (*P* = 0.896), and there was no significant difference between patients in MGFA grade III and IVa (*P* = 0.052). MG-ADL (*P* < 0.001) and MGC (*P* < 0.001) were positively correlated with MG-QOL15. Men had higher MG-QOL15 than women (*P* = 0.094), and LOMG had higher MG-QOL15 than EOMG (*P* = 0.072). Multivariate linear regression identified that higher MG-ADL (*P* < 0.001), higher MGC (*P* = 0.02), and poor employment status (*P* = 0.045) were independently associated with higher MG-QOL15.

**Conclusion:**

Having trouble using the eyes accounted for the highest score in MG-QOL15, eye symptoms affect QOL more than limb weakness in MG. Daily life activity, disease severity, and employment status were associated with patients' QOL. Adequate treatment should be applied to improve QOL, while mild symptoms can be accepted. Men and patients over the age of 50 years of onset may need more attention.

## Introduction

Myasthenia gravis (MG) is a rare autoimmune disease of the neuromuscular junction causing fluctuating muscle weakness. The incidence of MG in China is 0.68 per 100,000 person-years (1). According to different MG subgroups, the weakness can affect extraocular, facial, axial, limb, bulbar, and even respiratory muscles; therefore, muscle weakness and fatigability limit the autonomy of patients. Definitions of the MG subgroup are according to the presence of antibodies, symptoms, age at onset, thymus pathology, and distribution of weakness (ocular or generalized) (2, 3). Therapy includes symptomatic treatment using acetylcholinesterase inhibitors, thymectomy, and immunotherapy based on the MG subgroup (2). However, MG is a chronic disease for life, and long-term treatment often has many side effects (4).

Although the disability caused by MG is considered to be lower than other autoimmune diseases that affect the nervous system, such as neuromyelitis optica spectrum disorder and multiple sclerosis (5, 6), the aim of MG therapy should be clinical remission or mild symptoms with near-normal function and quality of life (QOL) not just focus on controlling muscle weakness symptoms (2). The goals for the treatment of MG are Minimal Manifestation Status (MMS), Pharmacologic Remission (PR), or Complete Stable Remission (CSR) (7, 8). Patients who achieve the goal can still have mild weakness, without functional limitations. Even so, 7% of patients with MG in China do not respond very well or are intolerant to conventional immunosuppressive therapy, meaning they require different types of biological therapy management (10). In addition, the side effects and psychosocial stress caused by long-term immunosuppressive therapy will likely affect the quality of patients' life. Therefore, measuring the QOL in patients with MG is important.

The 15-item Myasthenia Gravis Quality of Life (MG-QOL15) was developed by Burns et al. (11), in order to evaluate the QOL in patients with MG. MG-QOL15 has been used in several clinical trials as the outcome (12, 13). The MG-QOL15 has been successfully translated and validated into several languages including Chinese (14–16). Patient-reported outcome measures are also gaining increasing attention, both for MG and other chronic inflammatory diseases (17), such as MG activities of daily living (MG-ADL). The previous study suggests that more severe symptoms are associated with QOL scores (18–20). Such that, patients with refractory generalized MG rather than ocular MG may have lower QOL (21). Poor QOL was also related to depression in patients with MG (22). Age and age of onset alongside other factors including body mass index (BMI), type of work, education status, physical activity, and gender can also influence QOL in some populations with MG (18, 23). However, few studies about the QOL in MG have been undertaken in China (24–26). Therefore, this study aims to explore the QOL in patients with MG and the factors associated with QOL.

## Materials and methods

### Study design and patients

This observational study included continuous patients with a confirmed diagnosis of MG at the First Affiliated Hospital of Fujian Medical University between January 2020 and March 2022. The inclusion criteria were as follows: (1) age >14 years old; (2) confirmed MG diagnosis based on a combination of clinical history, physical examination, fatigue test results, and positive response to prostigmin test, or decremental response to repetitive nerve stimulation, or the presence of anti-acetylcholine receptors (AChR), or muscle-specific tyrosine kinase protein (MuSK) antibodies; (3) agreement to attend assessments; and (4) the medical records and evaluation were complete. Patients with Myasthenia Gravis Foundation of America (MGFA) clinical classification (8) of IVb and V were excluded. This study was approved by the Ethic Review Form for the Branch for Medical Research and Clinical Technology Application, Ethics Committee of First Affiliated Hospital of Fujian Medical University [2020] 243. All patients included have signed the written informed consent.

### Questionnaire and medical examination

The current severity of the disease, QOL, and serological status of all patients was evaluated by personal face-to-face interviews and medical examination by the same neurologist.

The current severity of the disease was clinically assessed using the MGFA grade (8), MG-ADL score, myasthenia gravis composite (MGC) score, and MGFA postintervention status. The MGFA grade was classified as Remission, I, II, III, and IVa (8). The MG-ADL includes eight items that can evaluate daily living functions. The MGC includes 10 items that can measure the clinical status of patients with MG (9). The MGFA postintervention status was classified as CSR, PR, and MMS (8). All the items used to assess the current severity of MG were evaluated as MG-QOL15 assessment at the same time.

The quality of life of the patients was assessed with the MG-QOL15, a self-administered disease-specific questionnaire consisting of 15 items. Response to each item was scored on a scale of “0,” “1,” “2,” “3,” and “4” representing “not at all,” “a little bit,” “somewhat,” “quite a bit,” and “very much,” respectively (10).

The serological status includes AchR-Ab and MuSK-Ab. AchR-Ab was tested using the radioimmunoprecipitation assay (RIA) or enzyme-linked immunosorbent assay (ELISA) technique or the cell-based assay (CBA) technique. MuSK-Ab was tested using the CBA technique.

The patients were stratified into disease subgroups according to (1) age at disease onset: <50 years was early-onset MG (EOMG) and ≥50 years was late-onset MG (LOMG); (2) distribution of muscle weakness: ocular myasthenia gravis (OMG), generalized myasthenia gravis (not involving bulbar weakness, GMG), or bulbar myasthenia gravis (involving bulbar weakness, BMG); (3) serological status: AchR-Ab and Musk-Ab; and (4) current severity of the disease (2, 3).

The clinical and demographic characteristics (including age, gender, age of onset, duration, education, employment state, marital status, skeletal muscle affected, thymic histology, and current treatment methods) were collected from electronic medical records.

### Statistics analysis

SPSS version 26.0 (IBM Corp., USA) and Prism 9 (GraphPad, USA) were used for statistical analysis. Continuous data that conformed to normal distribution were expressed as means ± standard deviation (SD) and compared by independent *t*-test. Continuous data that had skewed distribution were expressed as median (range) and compared by Mann–Whitney test. Categorical data were expressed as *n* (%) and compared by χ^2^ test. To compare continuous variables between more than two subgroups, one-way ANOVA, least significant difference (LSD), and Student–Newman–Keuls (SNK) tests were used for normally distributed data. While Kruskal–Wallis H test and Bonferroni test were used for non-normally distributed data. Correlations were assessed using Pearson's correlation coefficients or Spearman's correlation coefficients according to the data distribution. Multivariate linear regression analysis was used to explore the association between clinical characteristics and QOL (Supplementary Table 1).

## Results

**Figure 1:**
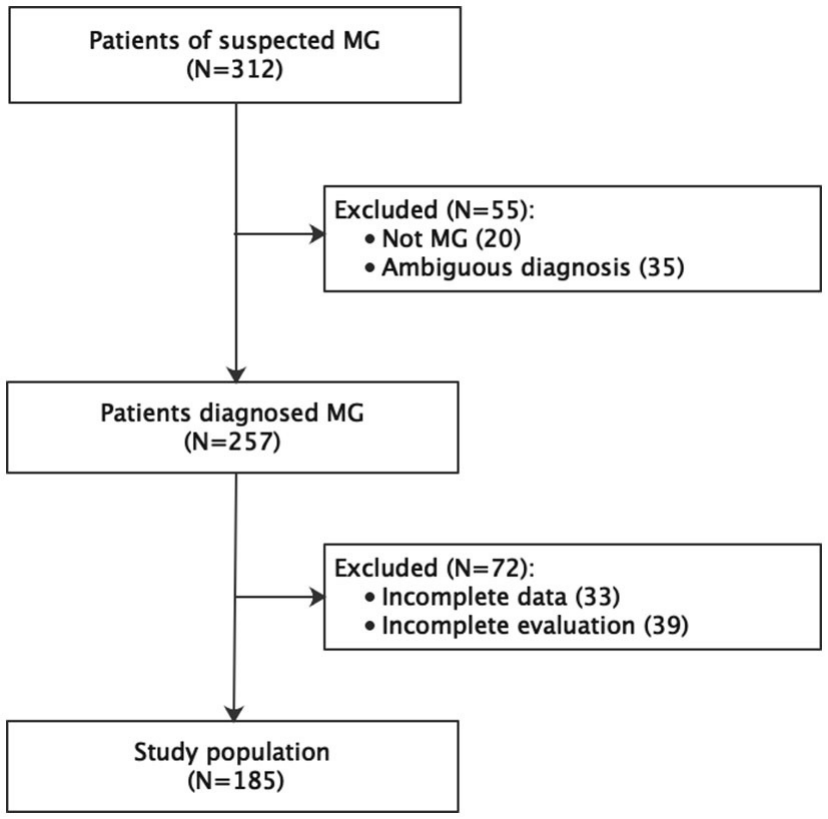
Recruitment flowchart. MG, myasthenia gravis.

A total of 312 patients were considered for inclusion in the study. Of them, 55 were excluded for an ambiguous diagnosis (*n* = 20) or not being diagnosed with MG (*n* = 35). A further 72 patients were excluded for incomplete data (*n* = 33) or incomplete evaluation (*n* = 39). Therefore, the final study population comprised 185 patients (Figure 1). The age at onset of the disease was 38.3 ± 17.9 years and at inclusion was 45.2 years (14–77), and the disease duration at inclusion was 87.9 months (0–672).

**Figure 2:**
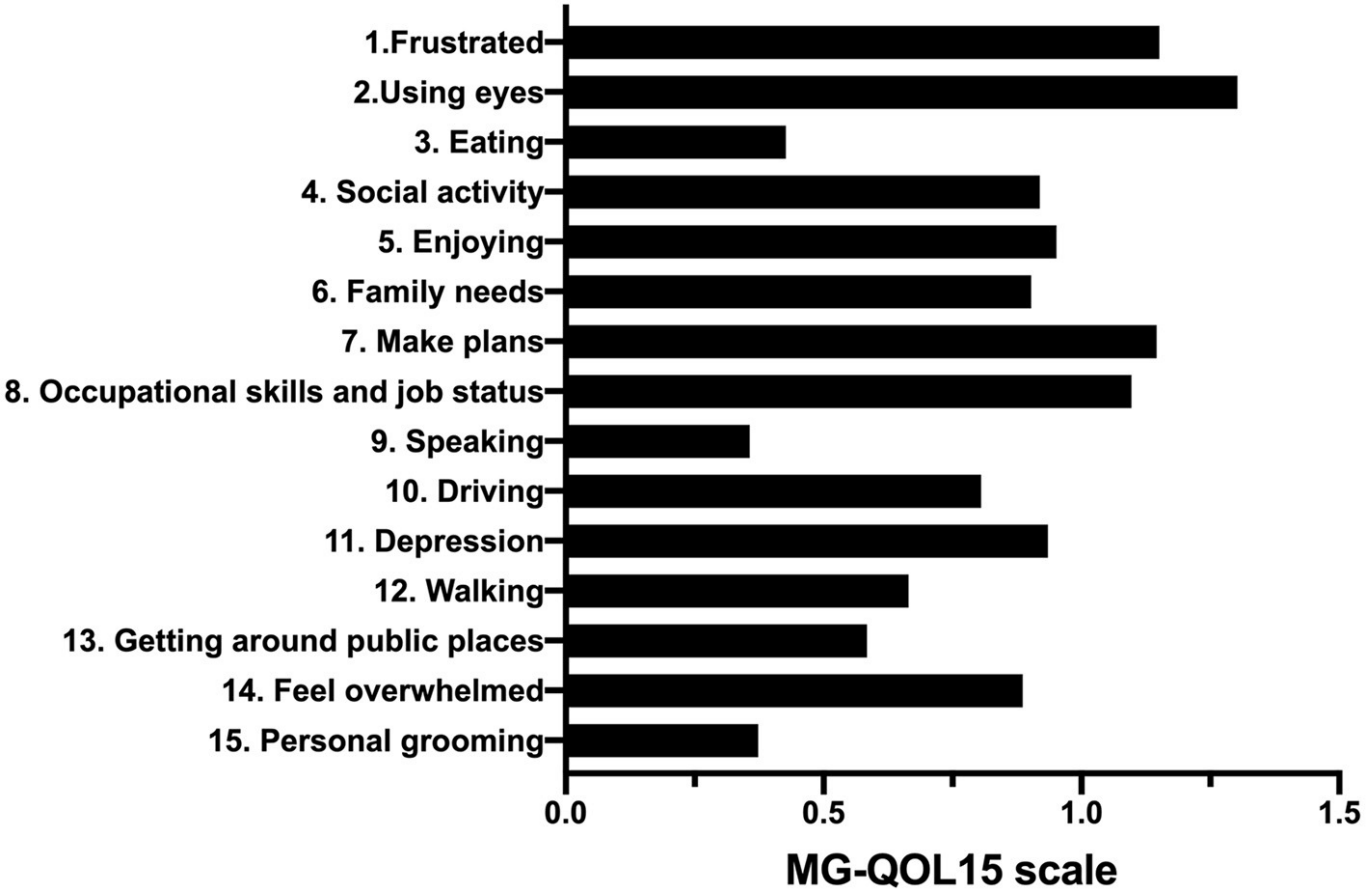
The 15 items of the MG-QOL15 scale. MG-QOL15, the 15-item Myasthenia Gravis Quality of Life.

The internal consistency (Cronbach's alpha = 0.937) and construct validity (KMO = 0.917, *P* < 0.001) of MG-QOL15 were good in this study. The mean MG-QOL15 score in this study was 12.5 (0–58). The item “have trouble using my eyes” was the highest scoring item in both ocular and generalized patients with MG (Figure 2). The median MG-ADL was 2.91 (0–20), and the median MGC was 3.71 (0–31).

**Figure 3:**
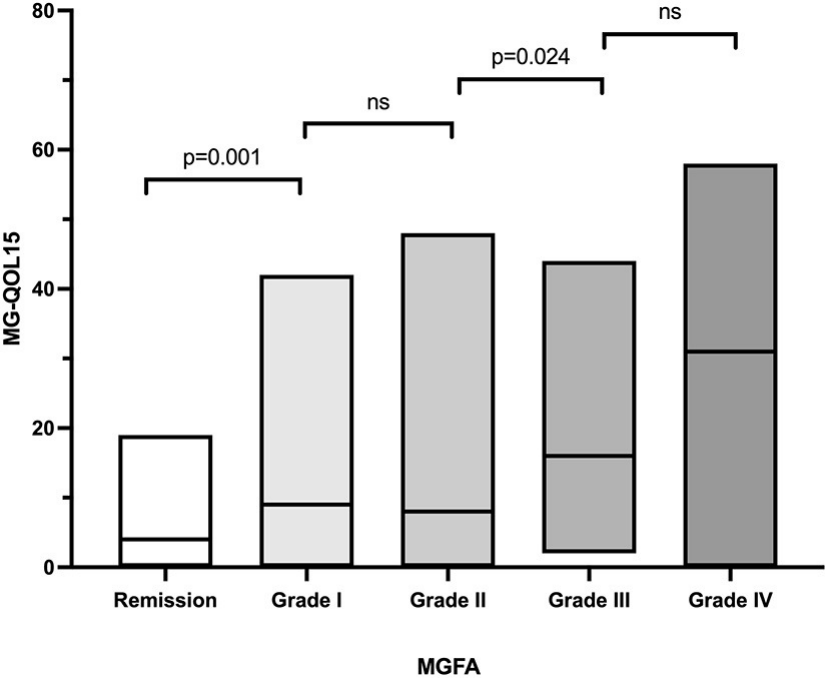
The MGFA grade and MG-QOL15. ns, no significance; MGFA, Myasthenia Gravis Foundation of America; MG-QOL15, the 15-item Myasthenia Gravis Quality of Life.

The MG-QOL15 score was significantly different among patients with OMG (9.2 ± 9.4, *n* = 63), GMG (9.0 ± 8.8, *n* = 22), and BMG (15.4 ± 14.2, *n* = 100) (*P* = 0.018). Patients with BMG had higher MG-QOL15 scores than OMG (*P* = 0.001) and GMG (*P* = 0.009), but there was no significant difference between OMG and GMG (*P* = 0.467). The MG-QOL15 score was significantly lower in patients who had undergone thymectomy (9.7 ± 9.8, *n* = 58) compared to those who had not (13.8 ± 13.4, *n* = 127, *P* = 0.022). The MG-QOL15 score was significantly different among patients with current MGFA grade of Remission (5.2 ± 5.4, *n* = 41), I (11.3 ± 10.0, *n* = 61), II (11.6 ± 11.1, *n* = 40), III (18.1 ± 12.1, *n* = 29), and IVa (30.1 ± 20.0, *n* = 14) (*P* < 0.001). Remission patients had lower MG-QOL15 scores than patients in MGFA grade I (*P* = 0.001), and patients in MGFA grade II had lower MG-QOL15 than patients in MGFA grade III (*P* = 0.024). But there was no significant difference between patients in MGFA grade I and II (*P* = 0.896), and there was no significant difference between patients in MGFA grade III and IVa (*P* = 0.052; Table 1, Figure 3). Men had higher MG-QOL15 scores than women (14.4 ± 13.7 vs. 11.3 ± 11.6, *P* = 0.094), and LOMG had higher MG-QOL15 score than EOMG (15.2 ± 14.3 vs. 11.3 ± 11.5, *P* = 0.072).

**Table 1:** Patient characteristics.

**Characteristics**	***N*** **(%)**	**MG-QOL15**	***P-*** **value**
**Gender**			
Male	72 (38.9%)	14.4 ± 13.7	0.094
Female	113 (61.1%)	11.3 ± 11.6	
**Disease onset**			
EOMG	128 (69.2%)	11.3 ± 11.5	0.072
LOMG	57 (30.8%)	15.2 ± 14.3	
**Serological status**			
AChRAb+	120 (64.9%)	13.3 ± 12.3	0.385
MuSKAb+	2 (1.1%)	9.0 ± 7.1	
AChRAb– MuSkAb–	33 (17.8%)	10.7 ± 12	
No data	30 (16.2%)	11.5 ± 14.4	
**Distribution of weakness**			
OMG	63 (34.1%)	9.2 ± 9.4	0.018
GMG	22 (11.9%)	9.0 ± 8.8	
BMG	100 (54%)	15.4 ± 14.2	
**Thymectomy**			
Yes	58 (31.4%)	9.7 ± 9.8	0.022
No	127 (68.6%)	13.8 ± 13.4	
**Education**			
Elementary	43 (23.2%)	15.7 ± 14.3	0.343
Secondary	100 (54.1%)	12 ± 12.4	
University degree or above	42 (22.7%)	10.4 ± 10.5		
**Marital status**			
Married	141 (76.2%)	13.1 ± 12.8	0.682
Not married	34 (18.4%)	11.3 ± 12.5	
Divorced	10 (5.4%)	8.4 ± 6.4	
**Employment status**			
Employed	78 (42.2%)	9.8 ± 8.9	0.218
Retired by age	23 (12.4%)	12 ± 13.3	
Unemployed due to MG	34 (18.4%)	16.4 ± 15.2	
Unemployed	50 (27.0%)	14.3 ± 14.3	
**Current MGFA grade**			
Remission	41 (22.2%)	5.2 ± 5.4	< 0.001
I	61 (33.0%)	11.3 ± 10	
II	40 (21.6%)	11.6 ± 11.1	
III	29 (15.7%)	18.1 ± 12.1	
IVa	14 (7.6%)	30.1 ± 20	
Total	185	12.5 ± 12.5	

MG-QOL15, The 15-item Myasthenia Gravis Quality of Life; EOMG, early-onset myasthenia gravis; LOMG, late-onset myasthenia gravis; AChRAb, acetylcholine receptor antibody; MuSKAb, muscle-specific kinase antibody; OMG, ocular myasthenia gravis; GMG, generalized myasthenia gravis; BMG, bulbar myasthenia gravis; MGFA, Myasthenia Gravis Foundation of America.

**Figure 4:**
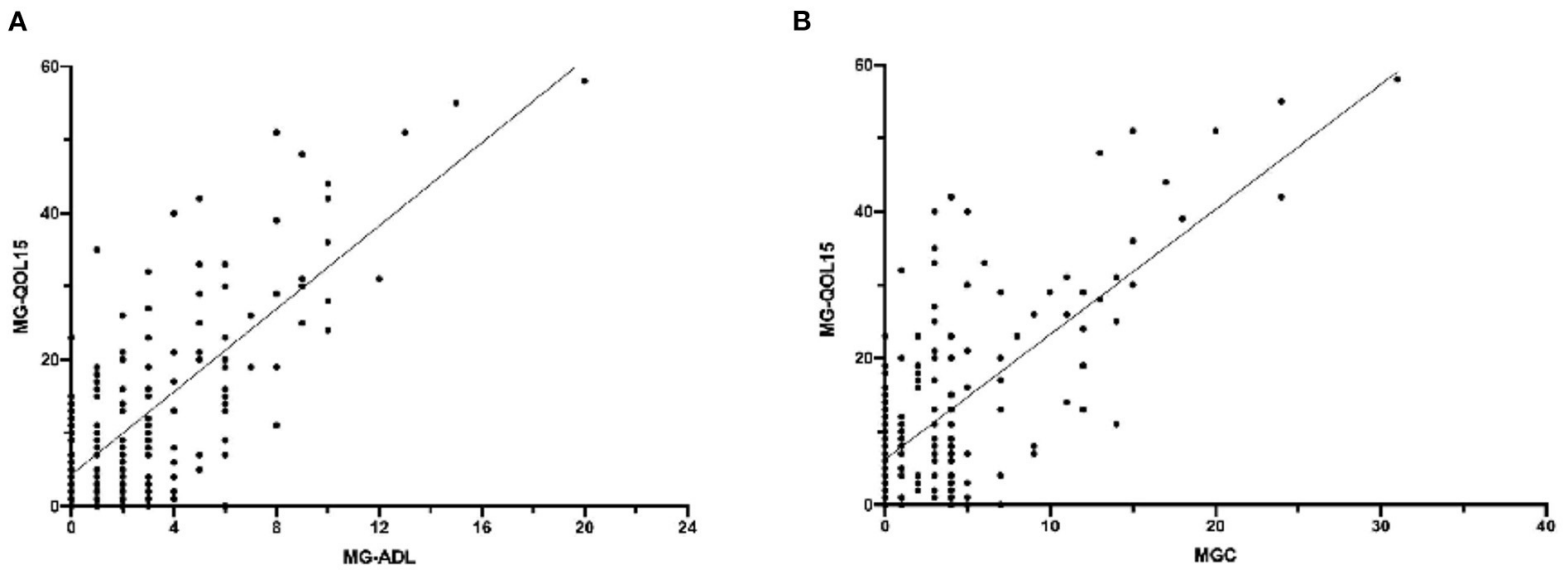
The correlation of MG-QOL5 with MG-ADL and MGC. **(A)** MG-ADL was positively correlated with MG-QOL15, *P* < 0.001, *r* = 0.734. **(B)** MGC was positively correlated with MG-QOL15, *P* < 0.001, *r* = 0.696. MG-QOL15, the 15-item Myasthenia Gravis Quality of Life; MG-ADL, myasthenia gravis activities of daily living; MGC, myasthenia gravis composite.

A total of 54 patients had attained CSR, PR, or MM status. Notably, 34 patients attained remission, while 20 patients attained MM. There was no statistically significant difference in total MG-QOL15 or every single item between patients with remission and patients with MM (Table 1). MG-ADL (*r* = 0.734, *P* < 0.001) and MGC (*r* = 0.696, *P* < 0.001) were positively correlated with MG-QOL15 (Figure 4). However, there were no significant correlations between MG-QOL15 and age at onset (*P* = 0.082), age (*P* = 0.172), or duration (*P* = 0.384).

Multivariate linear regression analysis showed that higher MG-ADL (β = 0.509, *P* < 0.001), higher MGC (β = 0.248, *P* = 0.02), and poor employment status (retired by age compared to unemployed, β = −0.125, *P* = 0.045) were independently associated with higher MG-QOL15 (Table 2).

**Table 2:** Multivariate linear regression analysis.

**Characteristics**	**Standardized β**	***P-*** **value**	**VIF**
Gender	−0.021	0.699	1.235
Age at onset (years)	0.385	0.173	34.53
Disease onset (EOMG/LOMG)	−0.095	0.269	3.176
Academic levels	−0.019	0.755	1.659
Age at inclusion (years)	−0.182	0.466	26.949
Durations (months)	0.25	0.111	10.62
MG-ADL	0.509	< 0.001	4.326
MGC	0.248	0.02	4.893
**Employment (vs. unemployed)**			
Employed	−0.134	0.082	2.555
Retired by age	−0.125	0.045	1.648
Unemployed due to MG	0.081	0.206	1.754
**Marital status (vs. not married)**			
Married	0.014	0.837	2.087
Divorced	−0.037	0.526	1.469
Thymectomy	0.089	0.103	1.271
Distribution of weakness	−0.004	0.945	1.487
Current MGFA grade	0	0.999	1.976

R = 0.784, R2 = 0.614, Adjusted R2 = 0.578, D-W = 1.951, *P* < 0.001.

MGFA, Myasthenia Gravis Foundation of America; MG-ADL, myasthenia gravis activities of daily living; MGC, myasthenia gravis composite.

## Discussion

This study showed the median MG-QOL15 was 12.5 (0–58), and the highest scoring item was “have trouble using my eyes” in both ocular and generalized patients with MG. Daily life activity, disease severity, and employment status were associated with patients' QOL. The result may provide a theoretical basis for the study on QOL of patients with MG.

One major finding of this study suggests that the QOL of patients with MG is affected by the severity of the disease. Worse MGFA grade was related to higher MG-QOL15, which is similar to previous studies (14, 18, 26). There was another study that suggested that refractory MG rather than OMG may have lower QOL (21), in that study, they did not distinguish between GMG and BMG. Furthermore, our results showed that the MG-QOL15 increased with increasing MG-ADL and MGC, indicating that controlling symptoms are key to improving QOL.

MG-ADL and MGC can reflect the severity of the disease and have been the primary and key secondary endpoints in many clinical trials (13, 27, 28). Multivariate linear regression analysis in this study showed that MG-ADL and MGC were independently associated with MG-QOL15. However, there was no statistically significant difference in MG-QOL15 between patients in remission and those with MM that result provides support for the therapeutic goal of MG being no functional limitation symptoms rather than no symptoms at all. It is interesting to note that there was no statistically significant difference in MG-QOL15 between MGFA grade I and grade II patients, which seemed to support the finding that eye symptoms affect QOL more than limb weakness in MG.

Most patients with MG can control symptoms with current treatments, in this study, many still had mild symptoms which did not affect their QOL. The goal of care in MG is to achieve a maximal clinical benefit on low-dose immunosuppressants (7), the Myasthenia Gravis Status and Treatment Intensity (MGSTI) score combined with the modified MGFA post intervention status (PIS) and immunosuppressant doses have been designed to be an endpoint in clinical trials (29). Patients who had undergone thymectomy had lower MG-QOL15 than those who had not undergone thymectomy in our study, this seemed to support the benefits of thymectomy (30). Previous studies have found that lack of employment may affect the quality of life in patients with MG (22, 31). However, our study found that retirement by age is a negatively correlated factor in MG-QOL15, indicating that patients who had a normal working life seemed to have a better QOL. In contrast to some other studies, this study did not find statistically significant differences in QOL with gender, age, age of onset, or education status (18, 25). This may be due to differences in the patient populations or the small sample size. It seems that men and LOMG might have worse QOL, even if not significant, which indicated that men and patients over the age of 50 years of onset may need more attention.

Coronavirus disease 2019 (COVID-19) is a SARS-CoV-2 infection that was declared a pandemic by the WHO in March 2020. Neuromuscular manifestations occurred frequently and might appear as the first symptom of COVID-19 (32), and it significantly impacted MG patients with an increase in mortality due to respiratory sequelae (33). Although the subjects included in this study did not experience SARS-CoV2 infection, the pressing issues caused by COVID-19 may still affect the QOL of patients with MG.

This study has some limitations. First, this is a single-center study which may, therefore, be subject to selection bias. Second, not all of the patients included completed the AChR-ab and MuSK-ab test, which may cause bias in the proportion of different serological statuses. Third, because there is some overlap among MG-QOL15, MGC, and MG-ADL, multicollinearity existed in the multivariate linear regression model.

## Conclusion

In summary, having trouble using eyes accounted for the highest score in QOL. The QOL of patients with MG might be associated with daily life activity, disease severity, and employment status, but mild symptoms did not affect the QOL. The men and patients over the age of 50 years of onset may need more attention.

## Data availability statement

The raw data supporting the conclusions of this article will be made available by the authors, without undue reservation.

## Ethics statement

The studies involving human participants were reviewed and approved by Ethic Review Form for Branch for Medical Research and Clinical Technology Application, Ethics Committee of First Affiliated Hospital of Fujian Medical University [2020] 243. Written informed consent to participate in this study was provided by the participants' legal guardian/next of kin.

## Author contributions

XW: writing and editing. RL: data curation and writing. XY: data statistics. NW: conceptualization and methodology. All authors contributed to the article and approved the submitted version.
