# Supplementary material for: Reduced quality of life in myasthenia gravis patients: A study on 185 patients from China
Source: Front Neurol. 2023 Jan 12;13:1072861. doi: 10.3389/fneur.2022.1072861 (PMC9878687; doi:10.3389/fneur.2022.1072861)
Supplement: Supplementary file 1 [file Table_1.docx]

**Supplementary Table 1. Code for characteristics included in multivariate linear regression analysis**

| Characteristics | Code |
| --- | --- |
| MGFA Clinical Classification | Continuous data |
| Gender |  |
| Male | 1 |
| Female | 2 |
| Disease onset |  |
| EOMG | 1 |
| LOMG | 2 |
| Distribution of weakness |  |
| OMG | 1 |
| GMG | 2 |
| BMG | 3 |
| Thymectomy |  |
| Yes | 1 |
| No | 2 |
| Education |  |
| Elementary education | 1 |
| Secondary | 2 |
| University degree or above | 3 |
| Marital status |  |
| Married | V1=1，V2=0 |
| Not married | V1=0，V2=0 |
| Divorced | V1=0，V2=1 |
| Employment |  |
| Employed | F1=1，F2=0，F3=0 |
| Retired by age | F1=0，F2=1，F3=0 |
| Unemployed to MG | F1=0，F2=0，F3=1 |
| Unemployed | F1=0，F2=0，F3=0 |

MGFA: Myasthenia Gravis Foundation of America; EOMG: early-onset myasthenia gravis; LOMG: late-onset myasthenia gravis; OMG: ocular myasthenia gravis; GMG: generalized myasthenia gravis; BMG: bulbar myasthenia gravis; MG: myasthenia gravis.
